# The Involvement of Cholinergic Neurons in the Spreading of Tau Pathology

**DOI:** 10.3389/fneur.2013.00074

**Published:** 2013-06-17

**Authors:** Diana Simón, Félix Hernández, Jesús Avila

**Affiliations:** ^1^Centro de Biología Molecular Severo Ochoa, CSIC-UAMMadrid, Spain; ^2^Centro de Investigación Biomédica en Red sobre Enfermedades NeurodegenerativasMadrid, Spain

**Keywords:** tau, muscarinic receptors, cholinergic neurons

## Abstract

Long time ago, it was described the selective loss of cholinergic neurons during the development of Alzheimer disease (AD). Recently, it has been suggested that tau protein may play a role in that loss of cholinergic neurons through a mechanism involving the interaction of extracellular tau with M1/M3 muscarinic receptors present in the cholinergic neurons. This interaction between tau and muscarinic receptors may be a way, although not the only one, to explain the spreading of tau pathology occurring in AD.

## Introduction

Alzheimer disease (AD) is characterized by the presence of two aberrant structures in the brain of the patients, senile plaques and neurofibrillary tangles, together with a clear loss of neurons that results, with the development of the disease, in a decrease in brain volume. Senile plaques are extracellular deposits of beta amyloid peptide (Masters et al., 1985) whereas tangles are composed of intracellular filamentous (paired helical filaments, PHFs) aggregates of tau protein in phosphorylated form (Grundke-Iqbal et al., 1986). Thus, in AD there are amyloid and tau pathologies. We will focus on tau pathology, but first we will comment on tau protein.

## Tau Protein

Tau protein was first described as a brain microtubule associated protein (Weingarten et al., 1975). cDNA tau was isolated later on from a mouse brain library, cloned, and sequenced (Lee et al., 1988). Studies in human brain samples showed that six different tau isoforms are expressed in the central nervous system (CNS) (Goedert et al., 1989, 1992a) whereas in peripheral nervous system a characteristic big tau isoform can be found (Goedert et al., 1992a,b).

The presence or absence of exons 2, 3, and 10 (Himmler, 1989) determines the presence of CNS tau isoforms. Exon 2 can appear alone in a tau isoform but exon 3 never appears independently of exon 2 (Andreadis et al., 1995). On the other hand there are tau isoforms with or without exon 10. The combination of all of these features results in the appearance of six tau isoforms. Exons 2 and 3 are located at the N-terminal region whereas exon 10 is presented close to the C-terminal end.

By comparing tau proteins from different organisms (Nelson et al., 1996), several variations were found at the N-terminal half of the protein whereas the C-terminal half of the molecule is well conserved among the different tau proteins (Nelson et al., 1996; Leon-Espinosa et al., 2013). The previous structural characteristics indicated for tau proteins could be related to their functions. These functions may be related to its subcellular localization and their binding to other proteins. The best tau-binding protein is tubulin, the main component of microtubules. This binding takes place through the conserved C-terminal half of tau molecule (Lee et al., 1988). On the other hand, tau can bind to other proteins through its N-terminal half. Among those proteins may be those containing SH3 domains (for a review, see Avila et al., 2004). More recently, it has been indicated that tau sequence RTPPKSP could bind to the SH3 domain of protein FYN (Bhaskar et al., 2010; Ittner et al., 2010). This tau sequence could also be involved in the interaction of tau with the protein phosphatase PP2A/B alpha (Sontag et al., 2012).

## Subcellular Localization of Tau Protein

Tau protein is mainly located at the cytoplasm of neurons where it binds to microtubules. The binding of tau to microtubules results in the stabilization of the polymers (Drubin and Kirschner, 1986), suppression of microtubule dynamics, and promotion of the formation of cytoplasmic extensions (Caceres and Kosik, 1990). At the cytoplasm, tau can bind to other proteins like kinases, phosphatases, acetylases, or deacetylases resulting, after those interactions, in a modified protein, which determines the subsequently binding of tau to other proteins. Other tau-binding protein is calmodulin, a protein that could be located at the cytoplasm or at the nucleus. Recently, it was suggested that tau could do a partial trapping of calmodulin at the cytoplasm decreasing the presence of calmodulin on nucleus and thus regulating, in this way, its activity as a co-transcription factor (Barreda and Avila, 2011).

Tau protein can also be present in the cell nucleus, although it has not yet been identified a nuclear transport signal on tau protein. Sometime ago, it was described that tau phosphorylation could be required for its transport to the nucleus (Greenwood and Johnson, 1995). Little is known about the function of nuclear tau but we know that tau binds to DNA (Corces et al., 1980). Tau could interact with nucleolar organizer regions of acrocentric chromosomes in some non-neuronal cells (Thurston et al., 1996). *In vitro*, tau prevents DNA replication but not transcription (Li et al., 2005) and it may behave like a histone-like protein. A role in neuronal DNA protection has also been proposed (Sultan et al., 2011).

Tau has also been found associated with some membrane components, like those involved in the formation of dendritic spines (Ittner et al., 2010) or at the presynaptic density (Moreno et al., 2011). The region of tau involved in the binding to the neuronal plasma membrane is the aminoterminal projection domain (Brandt et al., 1995). This tau region contains a proline rich sequence and it was described that phosphorylation of this sequence prevents the association of tau with plasma membrane (Arrasate et al., 2000). In the proline rich region there is a motif, PPXXP, that could bind to the SH3 domains present in some membrane associated proteins (Avila et al., 2004) and it may explain, at least in part, the interaction of tau protein with membrane.

## Tau Modifications

Two modifications, phosphorylation and aggregation, can regulate the interaction of tau with cytoplasmic, nuclear, or membrane components and it may result toxic for a cell. The largest CNS human tau isoform (Goedert et al., 1989) contains 79 potential serine/threonine sites that could be phosphorylated. Only few of those sites could be modified in normal conditions but in pathologies, like AD, this number could grow significantly (Hanger et al., 2009). Tau hyperphosphorylation could be toxic for a neuron as indicated by using cell culture and animal models (Brandt et al., 2005; Yoshiyama et al., 2007; Gomez de Barreda et al., 2010).

On the other hand, hyperphosphorylated tau can induce tau aggregation (Trojanowski and Lee, 1994; Alonso et al., 2001; Sato et al., 2002; Perez et al., 2003). The consequences of tau aggregation are a topic that remains in the field. It is discussed whether the presence of large tau aggregates could be toxic or beneficial for neurons (Bretteville and Planel, 2008). It has been shown that the number of extracellular tau aggregates (extracellular ghost tangles) is inversely proportional to the number of surviving neurons in the brain of AD patients. This observation is suggesting that at least some of the neurons that degenerate in the disease have previously developed tau aggregates (Bondareff et al., 1989). On the other hand, it has been proposed that the presence of tau aggregates could prevent the activation of cell promoting death molecules like caspase 3 (de Calignon et al., 2010). A possible explanation for those discrepancies could be found in the suggestion that the size of tau aggregates could be important for their toxic effect and that may be the small tau oligomers, and not large aggregates, the toxic agents (Maeda et al., 2007).

Also, overexpression of intracellular tau could be toxic for a cell (Andorfer et al., 2005). Since the levels of an intracellular protein are the consequence of its synthesis, degradation, and secretion, it was tested if an overexpression of intracellular tau could result in its secretion into microvesicles (Simon et al., 2012).

## Tau Pathology Spreading in the Presence or Absence of Neuron Death

Tau pathology usually starts at the entorhinal cortex and hippocampal region (Braak and Braak, 1991) and it may correlate with the loss of episodic memory occurring in the patients at the first stages of the disease. From the hippocampal region, tau pathology spreads to other brain areas and during the progression of the disease neurodegeneration and neuron death take place allowing that intracellular tau could be released to the extracellular space. Thus, intracellular and extracellular tau is present in neurodegenerative disorders like AD. Intracellular tau could be toxic due to its hyperphosphorylation level (Avila et al., 2004) or due to its aggregation (Bondareff et al., 1989; Gomez-Isla et al., 1997). However, it is discussed if larger aggregates like PHF, could be toxic (Cras et al., 1995; Avila, 2010; de Calignon et al., 2010).

## Extracellular Tau and Muscarinic Receptors

About extracellular tau, it has been suggested that once it is at the extracellular space it could become toxic for the surrounding neurons (Gomez-Ramos et al., 2006). Which is the mechanism for that toxicity will be commented below. However, an alternative way for tau pathology spreading, involving tau, has been reported. Thus, tau transmission from cell to cell could occur by exocytosis and endocytosis being not necessary neuron death (Clavaguera et al., 2009; Frost et al., 2009; de Calignon et al., 2012; Liu et al., 2012; Wu et al., 2012; Iba et al., 2013). On the other hand, to explain that the transmission could occur only in neurodegenerative disorders and not in a normal situation it has been proposed that aggregated tau is the toxic form for that spreading (Clavaguera et al., 2009; Frost et al., 2009; Iba et al., 2013). It is not clear if the endocytosis could take place in any cell type or if a specific cell receptor component is required. In this way, a specific transmission through synaptic connections has been proposed (de Calignon et al., 2012; Liu et al., 2012).

In the case of neuron death, intracellular tau is released to the extracellular space, and this extracellular tau could interact with surrounding neuronal cells and, as consequence of that, an increase in intracellular calcium can take place in those neurons (Gomez-Ramos et al., 2006). This increase in calcium could be due to calcium-permeable channels, to the activation of cell surface receptors coupled to calcium-influx or to calcium liberation from intracellular stores, induced by the activation of metabotropic receptors like muscarinic receptors (Gomez-Ramos et al., 2006). The published data have indicated that are, indeed, the muscarinic receptors, the ones involved in the interaction with extracellular tau and the responsible factors for raising intracellular calcium (Gomez-Ramos et al., 2008).

Muscarinic receptors subtypes have been classified in two groups: M1, M3, and M5, in one group, and M2 and M4 in the other group (Felder, 1995). The activation of M1 receptor group could activate phospholipase C, the release of inositol 1,4,5 triphosphate, and the subsequent mobilization of intracellular calcium (Felder, 1995). On the other hand, activation of M2 receptor group results in an inhibition of the intracellular levels of cAMP (Felder, 1995).

By using specific antagonists of either muscarinic receptors it was found that extracellular tau binds to M1 and M3 receptors and that it may explain the increase of intracellular calcium found in neuronal cells upon tau-binding (Gomez-Ramos et al., 2006, 2008, 2009). The region of human tau molecule involved in the binding to muscarinic receptors was described like that comprising residues 390–423 of the largest CNS human tau isoform (Gomez-Ramos et al., 2008). As consequence of that binding, tau protein could be or not endocytosed in a vesicle as M1 receptor does (Lameh et al., 1992).

## Binding of Modified Tau to Muscarinic Receptors

It was indicated that the toxicity of intracellular tau could be a consequence of its phosphorylation, or its aggregation. Thus, we have tested the consequences of phosphorylation or aggregation of extracellular tau on its interaction with muscarinic M1/M3 receptors. It was found that tau phosphorylation prevents the interaction of tau with muscarinic receptors. Also, it was described that extracellular phosphorylated tau is dephosphorylated by tissue-non-specific alkaline phosphatase (TNAP) and that this phosphatase, promotes the neurotoxic effect of extracellular tau (Diaz-Hernandez et al., 2010). The level of this phosphatase is increased in the brain of AD patients (Diaz-Hernandez et al., 2010).

It should be indicated that the level of both unphosphorylated and phosphotau, that could arise from dead neurons, are increased in the cerebrospinal fluid (CSF) of AD patients (Olsson et al., 2011).

Different levels of tau aggregation have been analyzed to study their interaction to muscarinic receptors. Thus, soluble tau containing monomers and small oligomers of tau, as well as purified larger tau aggregates (PHFs) have been tested. These studies have demonstrated that soluble tau but not PHFs interacted with muscarinic receptors (Gomez-Ramos et al., 2006).

## Consequences of the Interaction of Tau with Muscarinic Receptors

A consequence of that interaction is an increase in the level of intracellular calcium as previously described. A secondary effect of tau upon its binding to neuronal cells is to increase TNAP gene expression (Diaz-Hernandez et al., 2010). This effect could be the consequence of activation of DREAM, a transcription factor regulated by calcium (Carrion et al., 1999). However, this transcription factor probably is not involved in TNAP gene expression (Naranjo et al., unpublished results) and further analysis should be done to clarify the connection between calcium increase and TNAP gene expression.

## Other Consequences of Tau-Binding to Muscarinic Receptors

As previously indicated, upon interaction of tau with muscarinic M1/M3 receptors an increase in intracellular calcium takes place and some consequences of an increase in intracellular calcium could be an increase in secreted compounds. Preliminary experiments suggest an increase in the secretion of vesicles (containing flotilin) upon activation of M1/M3 receptors induced by tau protein, being that increase prevented by calcium chelators (Simon et al., unpublished results). Also, it has been described different affinities of tau and acetylcholine for M1/M3 receptors (Gomez-Ramos et al., 2009) and differences in the increase of intracellular calcium induced by ACh or tau protein through M1/M3 muscarinic receptors. Thus, for cell expressing M3 receptor, a minimum tau concentration of 50 pM was needed to find an increase in intracellular calcium while 5 nM ACh was required to have a similar effect (Gomez-Ramos et al., 2009). It was also found that a continuous increase in calcium level due to the presence of tau may result in cell death (Gomez-Ramos et al., 2009). Thus, it can be proposed that extracellular tau may promote cell death and it will result in the release of intracellular tau to the extracellular space and this new extracellular tau could again interact with other cells and, in this way, propagate neuron degeneration. This manner to propagate tau pathology may occur in AD or in other related pathologies (tauopathies). Little is known about how an increase of calcium mediated by the interaction of tau with muscarinic receptors could result in cell death. M1 and M3 receptors are coupled with G_q_/G_11_ proteins leading to activation of phospholipase C and an increase in the level of intracellular calcium. This calcium increase could activate some protein kinases, and these kinases could modify tau protein doing the protein toxic. In any case, further studies focused in the consequences of tau phosphorylation on neuron degeneration should be done.

## Tau Pathology Progression in Alzheimer Disease

Tau pathology spreading could involve (Gomez-Ramos et al., 2006), or not (Frost et al., 2009), neuronal death. It has been described that neuronal death and the presence of extracellular tau could be linked in some cases (Gomez-Ramos et al., 2006). In this way, an inverse correlation can be found between the number of extracellular tangles and the number of living neurons in the hippocampus (Bondareff et al., 1989). Also, extracellular tau is present in CSF of AD patients, suggesting neuronal death. As previously indicated, extracellular tau can have two different origins; one raised by exocytosis without cell death being this tau present, at least in part, in membrane vesicles and the other one, from neuronal death present in a naked form. The presence of both tau populations has been found in the CSF of AD patients. Tau present in vesicle particles was mainly found at the first stages of the disease whereas the amount of uncoated tau, in CSF, increases with the development of the disease (Saman et al., 2012).

We suggest that tau pathology spreading in cell culture, or *in vivo*, has a first step in which, probably, small tau oligomers specifically interact with neuron specific receptors. These receptors could be the M1/M3 muscarinic receptors although we cannot exclude other possibilities as an unspecific endocytosis pathway for tau internalization (Wu et al., 2012). Once tau is bound to the cell receptor, it could be endocytosed in a vesicle, a mechanism that occurs with ligands of M1/M3 receptors (Lameh et al., 1992), or simply, tau could promote the increase of intracellular calcium level and the appearance of second messengers or toxic compounds that will result in neuron death without being internalized. In the first case, the endocyted tau may interact with some cellular components, including tau itself, and it could be secreted uncoated or in a membrane vesicle (Lameh et al., 1992; Saman et al., 2012; Simon et al., 2012). These secreted vesicles could interact with other cells and be endocytosed in an unspecific way. In an alternative way, during the secretion, vesicles and cell membrane can be fused and uncoated tau protein (in aggregated or unaggregated form) could be released to the extracellular space where it can be toxic, upon interaction with muscarinic cell receptor. On the other hand, in other works have been reported that extracellular tau can induce intracellular tau aggregation and afterward the spreading of aggregated tau may occur in a prion-like manner (Clavaguera et al., 2009; Iba et al., 2013).

In summary, there are, at the present, different alternatives to explain tau pathology spreading in tauopathies like AD, a disease that long time ago was associated with severe loss of cholinergic markers in the brain (Davies and Maloney, 1976), and that such loss may be due to the toxic interaction of tau with muscarinic receptors.

## Conflict of Interest Statement

The authors declare that the research was conducted in the absence of any commercial or financial relationships that could be construed as a potential conflict of interest.
